# Stabilization of an archaeal DNA-sliding clamp protein, PCNA, by proteasome-activating nucleotidase gene knockout in *Haloferax volcanii*

**DOI:** 10.1111/j.1574-6968.2009.01547.x

**Published:** 2009-03-05

**Authors:** P Aaron Kirkland, Julie A Maupin-Furlow

**Affiliations:** Department of Microbiology and Cell Science, University of FloridaGainesville, FL, USA

**Keywords:** archaea, proteasome, halophile, DNA polymerase, sliding clamp, proteolysis

## Abstract

Many details of structure, function and substrate specificity of eukaryotic proteasomal systems have been elucidated. This information far-exceeds that available for the archaeal and bacterial counterparts. While structural and functional studies have provided some insight into the workings of prokaryotic proteasomes, the question of substrate targeting and global cellular influence remain largely unaddressed. In this communication, we report an over 720-fold increase in the half-life of the DNA-sliding clamp protein proliferating cell nuclear antigen after knockout of the *panA* gene, encoding a proteasome-activating nucleotidase A, on the chromosome of the halophilic archaeon *Haloferax volcanii*. This discovery marks the first identification of a protein stabilized by an archaeal proteasome mutation and provides a starting point for investigations into substrate recognition mechanisms. The findings also begin to address the functional role of proteasomal systems within the scope of the archaeal cell.

## Introduction

Proteasomes are energy-dependent proteases distributed in all three domains of life: *Bacteria, Archaea* and *Eucarya* (Volker & Lupas, 2002). Although proteasomes from these three domains share a common architecture and core molecular mechanism, little is known regarding the relatedness of their biological roles within the cell. In eukaryotes, proteasomes often require substrates to be covalently modified with ubiquitin before their destruction and are essential for catalyzing the precisely timed and rapid turnover of key regulatory and metabolic proteins as well as maintaining general protein quality control (Raasi & Wolf, 2007). In contrast, the biological roles of the proteasomal systems of archaea and bacteria are poorly understood.

Proteasomes are composed of a 20S core particle of α- and β-type subunits that associate with ATPase regulatory particles to facilitate protein unfolding and degradation (reviewed in Maupin-Furlow *et al.*, 2006). In archaea, proteasome-activating nucleotidase (PAN) proteins, closely related to the proteasomal regulatory particle triple-A type I proteins (Rpt) of eukaryotes, serve as ATPase components (Rabl *et al.*, 2008). Recently, we described the disruption of a gene on the *Haloferax volcanii* chromosome (*panA*) encoding a PAN protein present at high levels throughout exponential and stationary phase (Kirkland *et al.*, 2008). Disruption of this PAN paralog, PanA, has a pleiotropic effect on the (phospho)proteome of the cell and is anticipated to impede proteasome-mediated degradation of at least a subset of proteins identified by electrospray ionization tandem-mass spectrometry (MS/MS) to accumulate in the *panA* mutant compared with its parent strain (Kirkland *et al.*, 2008). This list includes a number of proteins with orthologs known to be targets of the ubiquitin–proteasome system in eukaryotes. In particular, our efforts for this study focused on the DNA-sliding clamp protein proliferating cell nuclear antigen (PCNA) of *H. volcanii*, which was identified as (phospho)proteome fractions enriched from lysate of *panA* mutant and wild-type cells using immobilized metal affinity chromatography (IMAC) (Kirkland *et al.*, 2008).

PCNA is a trimeric ring, which serves as a sliding DNA clamp to recruit leading and lagging strand DNA polymerases to the replication fork, aid in excision repair tasks and a myriad of other cellular functions (Moldovan *et al.*, 2007; Watson *et al.*, 2008). Previous reports in rice (*Oryza sativa*) (Yamamoto *et al.*, 2004) and humans (Wang *et al.*, 2006; Nikolaishvili-Feinberg *et al.*, 2008) suggest a role for the proteasome in PCNA turnover and, in a broader sense, reveal a more important global function for the proteasome in regulating DNA replication, recombination and/or repair. Indeed, phosphorylation of the tyrosine residue 211 of PCNA modulates its modification by polyubiquitination and ultimately its stability in human cancer cell lines (Wang *et al.*, 2006).

To assess whether PCNA is a target of the archaeal proteasome system, pulse-chase labeling with ^35^*S*-methionine and ^35^*S*-cysteine was coupled with immunoprecipitation and used to establish the rate of turnover of this protein in *H. volcanii* parent and *panA* mutant strains (wild type and Δ*panA*, respectively). Elongation initiation factor 2, α subunit (eIF2α) was included for comparison, because this protein also accumulated in proteasome-deficient cells of *H. volcanii*.

## Materials and methods

### Antibody preparation

To overcome interdomain differences in protein structure, custom antibodies were raised against the PCNA and eIF2α polypeptides of *H. volcanii*. In brief, the coding regions for PCNA (HVO_0175) and eIF2α (HVO_0699) were amplified from the *H. volcanii* genome (April 2007 version, http://archaea.ucsc.edu/) by PCR using Accuprime GC-Rich DNA polymerase (Invitrogen, Carlsbad, CA) and the following primer pairs: PCNA, 5′-TCCTCTT*CATATG*TTCAAGGCCATCGTGAG-3′ and 5′-AAA*CTCGAG*GTCACTCTGGATGCGCGGG-3′; eIF2α, 5′- TTGAGTT*CATATG*AAGTACAGCGGATGGCCTG-3′ and 5′-TTT*CTCGAG*CTCTTCGTCGCCGCTG-3′ (NdeI and XhoI sites in italics). The PCR fragments were cloned into the NdeI and XhoI sites of plasmid pET24b (Novagen, Madison, WI) to generate expression plasmids pJAM510 and pJAM511, respectively. The cloned DNA was sequenced to verify fidelity (UF ICBR DNA sequencing core, Gainesville, FL). Proteins were synthesized with C-terminal hexa-histidine tags (-His_6_) in recombinant *Escherichia coli* Rosetta (DE3) strains carrying these plasmids (pJAM510 and pJAM511). Proteins were purified from cell lysate by nickel column chromatography (Ni Sepharose 6 Fast Flow, Pharmacia) and reducing SDS-PAGE and used as antigens for the generation of polyclonal antibodies in rabbits, as previously described for other haloarchaeal proteins (Reuter *et al.*, 2004).

### Pulse-chase assay coupled with immunoprecipitation

*Haloferax volcanii* wild-type and *panA* deletion strains (DS70 and GG102, respectively) (see Wendoloski *et al.*, 2001; Kirkland *et al.*, 2008 for detailed genotypes) were grown in 200 mL minimal (Hv-Min) medium (Dyall-Smith, 2008) to an OD_600 nm_ of 0.4–0.6 (200 r.p.m., 42 °C). Cells were collected by centrifugation at 10 000 ***g*** for 15 min at 40 °C and resuspended in 80 mL of prewarmed (42 °C) Hv-Min medium. Cells were incubated at 42 °C for 1 h with shaking (200 r.p.m.). Radioactively labeled methionine and cysteine (500 μCi of ^35^*S*-methionine and ^35^*S*-cysteine mixture) (EXPRE^35^S^35^S Protein Labeling Mix, Perkin Elmer) were added to the culture and incubated for an additional 20 min (42 °C, 200 r.p.m.). Samples were divided into 8–10-mL fractions in 15-mL conical tubes. Translation was arrested immediately in one tube (0 min) by addition of 1 mL of ‘stop’ solution [22% (w/v) sodium azide, 100 μg mL^−1^ puromycin] and incubation on ice. The remaining seven samples were incubated with 1 mL of ‘chase’ solution (8 mg mL^−1^‘cold’ methionine and cysteine, 100 μg mL^−1^ puromycin, 1 mg mL^−1^ protease inhibitor cocktail) at 42 °C and 200 r.p.m. Each sample was arrested at appropriate time intervals (1, 3, 5, 10, 30 and 60 min and 12 h) by the addition of 1 mL of ‘stop’ solution and chilling on ice. Each 10-mL sample was collected by centrifugation at 8000 ***g*** at room temperature (RT) for 10 min. Pellets with *c*. 1 mL of medium were resuspended and repelleted in 2 mL microcentrifuge tubes at 14 000 ***g*** at RT for 5 min. Cell pellets were frozen at −80 °C for a maximum of 48 h before immunoprecipitation.

For immunoprecipitation, protein A–Sepharose beads (Pharmacia) [10 mg mL^−1^ phosphate-buffered saline (PBS) with 0.01% (w/v) sodium azide] were aliquoted into 1.8-mL Eppendorf tubes (75 μL per tube), washed once with cold PBS and resuspended in 1 mL of cold PBS. The beads were charged by addition of 10–12 μL of polyclonal antiserum for 4–12 h at 4 °C with continuous agitation and washed five times with cold PBS to remove unbound antibody. Cell pellets from pulse-chase labeling (described above) were prepared for immunoprecipitation by resuspension in 150 μL of denaturing lysis buffer [1% (w/v) sodium dodecyl sulfate (SDS), 50 mM Tris-Cl, pH 7.4, 5 mM EDTA, 10 mM dithiothreitol, 1 mM phenylmethylsulfonyl fluoride (PMSF) and 300 mM NaCl], boiling for 10 min and addition of 1.35 mL nondenaturing lysis buffer [1% (v/v) Triton X-100, 50 mM Tris-Cl, pH 7.4, 5 mM EDTA, 0.02% (w/v) sodium azide, 10 mM iodoacetamide, 1 mM PMSF, 300 mM NaCl]. The lysate was incubated with 500 U of benzonase (2 μL) (Sigma-Aldrich) at RT for 30 min with occasional mixing. Samples were clarified by centrifugation at 10 000 ***g*** for 5 min, and the clarified lysate was added to the charged beads. The lysate/bead mixture was incubated at 4 °C with rocking for 3 h. Beads were washed five times with immunoprecipitation wash buffer [0.1% (v/v) Triton X-100, 50 mM Tris-Cl, pH 7.4, 300 mM NaCl, 5 mM EDTA, 0.02% (w/v) sodium azide, 0.1% (w/v) SDS and 0.1% (w/v) deoxycholine] and one final time with cold PBS. SDS-reducing dye [20 μL of 100 mM Tris-Cl, pH 6.8, 10% (v/v) β-mercaptoethanol, 2% (w/v) SDS, 10% (v/v) glycerol and 0.6 mg mL^−1^ bromophenol blue] was added. Samples were boiled for 10 min and centrifuged at 14 000 ***g*** for 5 min to remove the beads. Proteins in the supernatant were separated by 12% SDS-polyacrylamide gel electrophoresis (PAGE) (200 V for 45 min), and their migration was compared with Precision Plus Protein dual color standards (BioRad). Gels were incubated in fixing solution (10% methanol, 7% acetic acid) for 30 min and an intensifier solution (1 M salicylic acid) for 1 h. Gels were washed with dH_2_O, dried under vacuum for 1.5–2 h, and exposed to radiographic film for a minimum of 10 days at −80 °C. Band intensity was quantified using a Versa Doc 1000 with quantity one v. 4 software (BioRad).

## Results and discussion

**Fig. 1:**
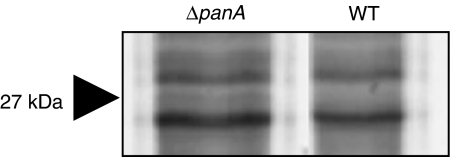
Comparison of IMAC-enriched proteins from cell lysate of *Haloferax volcanii* wild-type (WT) and *panA* deletion (Δ*panA*) strains. Proteins enriched from cell lysate by IMAC were separated by reducing 12% SDS-PAGE and stained with SYPRO Ruby as described previously (Kirkland *et al.*, 2008). Regions of reproducible and differential protein banding between the two strains such as the 25–30 kDa region shown were digested with trypsin and analyzed by MS/MS. PCNA DNA-sliding clamp protein of 27 kDa was identified by this semi-quantitative 1-D-gel approach.

**Fig. 2:**
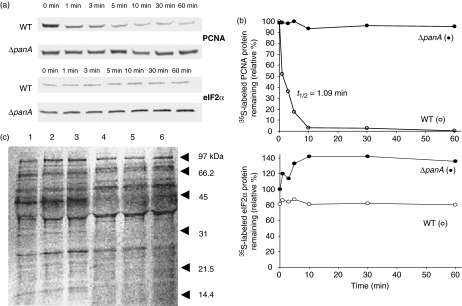
Comparison of the half-lives of PCNA DNA-sliding clamp protein and translation initiation factor eIF2α in *Haloferax volcanii* wild-type (WT, ○) and *panA* deletion (Δ*panA*, •) strains. (a) ^35^*S*-pulse-chase labeling was coupled with immunoprecipitation using anti-PCNA and anti-eIF2α polyclonal antibodies to monitor the respective half-lives of PCNA and eIF2α in wild-type and Δ*panA* mutant strains over a 12-h time course (with 12 h results similar to 1 h, data not shown). These data are representative of at least three independent experiments. (b) A graphical representation of the PCNA- and eIF2α-specific protein band intensities in percentages relative to 0 min for each protein, were used to calculate protein half-lives. (c) Similar overall levels of ^35^*S*-label were incorporated into the bulk-protein of wild-type (lanes 4–6) and Δ*panA* (lanes 1–3) strains. Total cell protein was radiolabeled with ^35^*S*-methionine and ^35^*S*-cysteine, separated by 12% SDS-PAGE and analyzed by autoradiography as described in Materials and methods.

The DNA-sliding clamp protein PCNA was identified by MS/MS analysis of IMAC fractions purified from cell lysate of an *H. volcanii panA* mutant as described previously (Kirkland *et al.*, 2008). IMAC fractions of the *panA* mutant (GG102) were compared with its parent strain (DS70) by 1-D gel electrophoresis, and regions of differential protein bands were excised and analyzed by MS/MS. The PCNA protein of 27 kDa was identified in the *panA* mutant IMAC fractions of gel slices in the 25–30-kDa region (Fig. 1), thus implicating PCNA as a potential substrate of the proteasomal system. To further investigate this finding, antibodies were raised against PCNA. Pulse-chase labeling with ^35^*S*-methionine and ^35^*S*-cysteine was coupled with immunoprecipitation to determine the rate of turnover of PCNA in *H. volcanii* parent (wild-type, WT) and *panA* mutant (Δ*panA*) strains (Fig. 2). Similarly, the half-life of eIF2α was analyzed for comparison. The PCNA- and eIF2α-specific protein bands detected by immunoprecipitation after pulse-chase were estimated at 27 and 30 kDa, respectively, based on migration in SDS-PAGE gels. The sizes of these bands were consistent with the molecular masses calculated for the respective polypeptides deduced from the *H. volcanii* genome sequence. Protein bands of similar molecular mass were not detected by immunoprecipitation using PCNA- or eIF2α-preimmune serum (data not shown).

Based on pulse-chase immunoprecipitation analysis, the PCNA protein had a surprisingly short half-life in wild-type cells of only *c*. 1 min with little protein remaining after 10 min (Fig. 2a and b). In contrast, no reduction in PCNA levels was detected over the course of 12 h for the *panA* mutant, which no longer synthesizes a functional PanA protein (Fig. 2a and b). Thus, the ^35^*S*-labeled PCNA was relatively stable in these *panA* mutant cells with a half-life >12 h. Interestingly, minute levels of PCNA persisted throughout the 12-h time course in the wild-type cells. Thus, although PCNA undergoes a high rate of protein turnover, low levels of this protein were retained within the cell for relatively long periods of time. Maintaining low levels of PCNA may reflect its critical role as a processivity-promoting factor in DNA replication and other functions that are essential for cell growth. Despite this observed, retention of PCNA protein in wild-type cells, it is clear that the levels of PCNA protein were reduced significantly (>90%) through a mechanism that requires synthesis of the PanA protein.

As a comparison, pulse-chase immunoprecipitation was also used to assess the half-life of eIF2α in wild-type and *panA* mutant strains (Fig. 2a and b). In contrast to PCNA, no appreciable reduction in the levels of eIF2α protein was observed for either strain over the 12-h time course. Although eIF2α was highly stable, the overall level of labeled eIF2α protein in cells possessing a fully functional proteasome was notably lower than that of the *panA* mutant. This difference is most likely due to a *panA*-dependent increase in the level of eIF2α-specific transcript and/or translation of the eIF2α-specific transcript, because similar levels of ^35^*S*-label were incorporated into the bulk protein of both strains as demonstrated by autoradiograph analysis of total cell protein separated by SDS-PAGE (Fig. 2c). These results are consistent with our previous MS/MS findings that the steady state levels of eIF2α protein are higher in the *panA* mutant compared with its parent.

In conclusion, the comparison of PCNA and eIF2α half-lives in the presence and absence of a functional proteasomal system (Δ*panA*) corroborates and builds upon our results, which identified, by MS/MS, enhanced levels of both of these proteins after perturbation of PanA function. It also enabled us for the first time to attribute these proteomic results to *panA*-dependent differences in PCNA protein half-life vs. the translational/transcriptional effects seen for eIF2α. These results suggest that, much like eukaryotes, archaeal proteasomes and/or their associated ATPase regulatory proteins (i.e. PanA) are intimately linked to regulating the levels and ultimately function of PCNA protein. Because PCNA orchestrates several functions associated with DNA replication, recombination and repair, the finding that PCNA is stabilized in a *panA* mutant may explain our previous results that this same mutation reduces the growth rate of *H. volcanii* (Kirkland *et al.*, 2008). Whether the *H. volcanii* PCNA protein is phosphorylated and this influences its stability, similar to the eukaryotic counterpart, remains to be determined. Our previous work demonstrated that PCNA was enriched from *H. volcanii* cell lysate by IMAC, a technique commonly used to purify phosphopeptides. Likewise, the Tyr211 residue shown to be phoshorylated in human and mouse PCNA orthologs (Wang *et al.*, 2006) is conserved in a wide group of eukaryotic and archaeal species, including *H. volcanii*. However, a specific site(s) of phosphorylation is yet to be identified for this or other archaeal PCNA proteins.

Together, these findings address the functional role of proteasomal systems within the scope of the archaeal cell and provide a starting point for investigations into mechanisms of substrate recognition. The apparent link of archaeal proteasomes with the DNA-sliding clamp protein, PCNA, points to future comparison of proteasome function in mixed populations to that of synchronized cells. Recent advances in the study of the haloarchaeal cell cycle (Baumann *et al.*, 2007) will facilitate this latter objective.

## Statement

Re-use of this article is permitted in accordance with the Creative Commons Deed, Attribution 2.5, which does not permit commercial exploitation.
